# [Retracted] Tripartite motif‑containing 14 regulates cell proliferation and apoptosis in cervical cancer via the Akt signaling pathway

**DOI:** 10.3892/mmr.2023.13100

**Published:** 2023-09-25

**Authors:** Wenjing Diao, Caiying Zhu, Qisang Guo, Yuankui Cao, Yu Song, Hua Feng, Jun Li, Xiaohong Xue, Pei Lu

Mol Med Rep 22: 5145–5154, 2020; DOI: 10.3892/mmr.2020.11634

Following the publication of this paper, it was drawn to the Editor's attention by a concerned reader that the GAPDH control western blotting data shown in Fig. 2B were strikingly similar to data appearing in different form in Fig. 1E in another article written by different authors at different research institutes [Liang T, Ye X, Liu Y, Qiu X, Li Z, Tian B and Yan D: FAM46B inhibits cell proliferation and cell cycle progression in prostate cancer through ubiquitination of β-catenin. Exp Mol Med 50: 1–12, 2018].

Owing to the fact that the contentious data in the above article had already been published prior to its submission to *Molecular Medicine Reports*, the Editor has decided that this paper should be retracted from the Journal. The authors were asked for an explanation to account for these concerns, but the Editorial Office did not receive a reply. The Editor apologizes to the readership for any inconvenience caused.

